# Molecular Insights into the Effect of Nitrogen Bubbles on the Formation of Tetrahydrofuran Hydrates

**DOI:** 10.3390/molecules27154945

**Published:** 2022-08-03

**Authors:** Xin Huang, Zhenchao Li, Le Zhang, Jiayuan He, Hailong Lu

**Affiliations:** 1 SINOPEC Petroleum Exploration and Production Research Institute, Beijing 102206, China; huangxin2020.syky@sinopec.com (X.H.); zhangle2017.syky@sinopec.com (L.Z.); hejy.syky@sinopec.com (J.H.); 2 Southern Marine Science and Engineering Guangdong Laboratory (Guangzhou), Guangzhou 511458, China; 3 Beijing International Center for Gas Hydrate, School of Earth and Space Sciences, Peking University, Beijing 100871, China; lizhenchao@pku.edu.cn

**Keywords:** hydrate, tetrahydrofuran, bubble, nolecular dynamics simulation

## Abstract

In this work, a molecular dynamics simulation was conducted to study the microscopic mechanism of how nitrogen bubbles affect the formation of THF hydrates at the molecular level. The results obtained reveal that the nitrogen bubble can promote the formation of THF hydrates. In the system with a nitrogen bubble, more THF-filled cages were generated, and the crystal structure was more orderly. The promotion of nitrogen bubbles on hydrate crystallization comes from the dissolution of nitrogen molecules. Some of dissolved nitrogen molecules can be enclosed in small hydrate cages near the nitrogen bubble, which can serve as stable sites for hydrate crystal growth, resulting in the fact that THF-filled cages connected with N_2_-filled cages are much more stable and have a long lifetime. The results in this work can help to understand the promotion effect of micro- and nano-air bubbles on the crystallization of THF hydrates.

## 1. Introduction

Clathrate hydrates are crystalline ice-like solids composed of water and guest molecules, with a host lattice formed by hydrogen-bonded water molecules [1]. Guest molecules can be gases or volatile liquids, such as methane, carbon dioxide or tetrahydrofuran (THF), which are enclosed within the polyhedral cages and interact with the host lattice via van der Waals interactions. As methane hydrate is a kind of potential source of energy with abundant resources in nature [2], much attention has been paid to the formation and aggregation of hydrates in porous sediment [3,4,5,6,7,8,9,10]. Furthermore, understanding of the hydrate formation mechanism is conducive to security strategies for flow assurance [11,12], and to apply the hydrates in energy storage [13,14], carbon dioxide capture and separation [15], and seawater desalination [16].

As THF can form clathrate hydrates by only controlling the temperature under the atmospheric pressure, THF hydrate is one of the most well-investigated clathrate hydrates, which is frequently used as a model for gas hydrates [17,18,19,20]. Currently, the formation behavior of THF hydrates has been investigated in both experimental [17,18,19,20] and simulation studies [21,22,23,24,25,26,27,28]. There are many factors that may affect hydrate formation, and one of the most important is micro- or nano-bubbles in liquid phase [29,30,31,32,33,34]. In nature, around gas chimneys and cold springs, it is a common phenomenon that clathrate hydrate will preferentially crystallize surrounding bubbles [35,36]. Many previous studies reveal that the presence of micro- and nano-bubbles can shorten the induction time for hydrate nucleation, and they attributed this phenomenon to the provision of gas sources and the high Laplace pressure of micro- and nano-bubbles [29,30,31,32,33,34]. In our prior experiment, in which we observed the crystallization of THF hydrates in the existence of micro- and nano-bubbles based on atomic force microscopy (AFM), it was found that even air bubbles can also promote the crystallization of hydrates [19]. However, due to the limitation of the AFM experimental test, the intrinsic mechanism is still unclear. 

Molecular dynamics (MD) simulation is a powerful tool that can be used to investigate the underlying physics of interesting experimental phenomena, which can overcome the shortcomings of uncontrollable factors in experiments. Therefore, there are more and more applications investigating the nucleation and growth mechanism of clathrate hydrates in recent years [21,22,23,24,25,26,27,28,37,38,39,40,41,42]. Several MD studies focus on the nucleation and growth mechanisms of clathrate hydrates containing THF [21,22,23,24,25,26,27,28]. The growth of THF hydrates was found to be faster at the (100) surface than at the (111) surface in the work of Nada [21]. Wu et al. studied the effect of THF concentration on the stability of THF hydrates, and investigated the nucleation and growth mechanism of methane-THF mixed hydrates [22,23,24]. Hydrate cages with THF molecules had short a lifetime, while they could be stabilized by neighboring hydrate cages with methane molecules. By using MD simulations, Yagasaki et al. found out that THF hydrates grow much slower than both ice and ethylene oxide hydrates [25,26]. Phan et al. confirmed that THF in stoichiometric concentrations would reduce carbon dioxide storage capacity [27]. Besides, solid media was also found to affect the formation of THF hydrates. By influencing the arrangement of THF and water molecules, the hydrophilic copper surface was found to be adverse for the formation of THF hydrates in the MD study of Ebrahimian et al. [28].

In this work, we studied the formation processes of THF hydrates with or without the presence of a nitrogen bubble using MD simulations, to reveal how air bubbles affect the formation processes of THF hydrates in our previous experimental study [19]. Since nitrogen is the main component of air, a nitrogen bubble was used for simplicity.

## 2. Computational Detail

**Figure 1:**
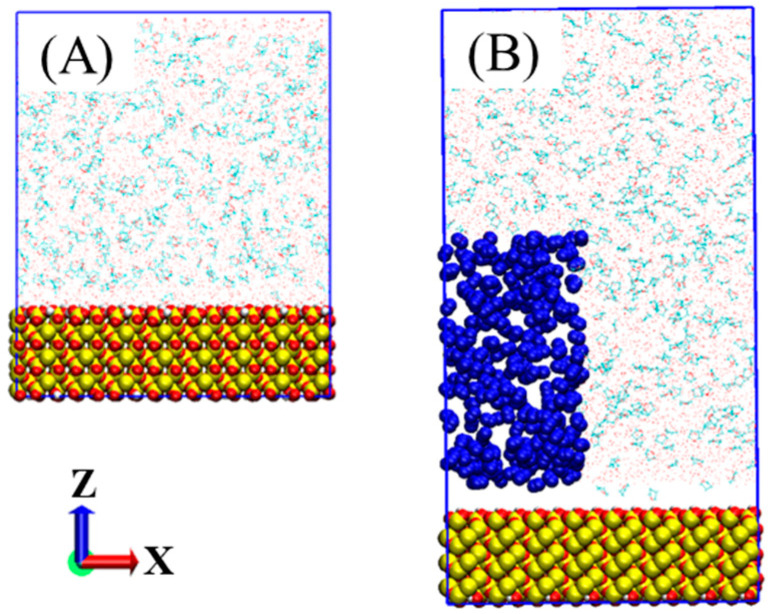
Models with (**B**) or without (**A**) the presence of a nitrogen bubble. The quartz plane is shown as balls (yellow for Si, red for O, and white for H), nitrogen atoms are represented as blue balls, THF and water molecules are described as lines (cyan for C, red for O, white for H).

To build the model without a nitrogen bubble, a 100% hydroxylated quartz crystal plane with the size of 6.8 × 6.8 × 2.1 nm^3^ was set as the substrate, and 6800 water and 400 THF molecules were placed in the 6.8 × 6.8 × 6.4 nm^3^ region above the quartz plane (Figure 1A). As for the model containing the nitrogen bubble, the box size was 6.8 × 6.8 × 13.0 nm^3^. The same quartz plane was set as the substrate, 300 nitrogen molecules were placed in a box of 3.0 × 6.8 × 6.0 nm^3^ above the quartz plane, and 6800 water and 400 THF molecules were used to fill the rest region (Figure 1B). The radio of THF and water molecules were equal to that in pure THF hydrates.

The initial configurations of each system were generated by the following step: Firstly, energy minimization was conducted for each model. Secondly, each system was relaxed at 300 K and 5 MPa for 5 ns. Thirdly, each system was relaxed for 1 ns at the pressure of 5 MPa and the temperature decreasing from 300 to 250 K at a rate of 0.05 K/ps. After the initial configurations of systems with or without the presence of a nitrogen bubble were obtained, MD simulations were conducted for each system at 250 K and 5 MPa for 2000 ns. Three repeated 1000 ns simulations for the system with bubbles were conducted, compared with those for the system without bubbles. The results of repeated simulations showed no significantly difference (Figure S1).

TIP4P-ice model [43,44], TraPPE force field [45], CGenFF [46,47], and CLAYFF [48] were used to describe water, N_2_, THF, and the quartz surface respectively. As listed in Table 1, the interaction Lennard-Jones parameters between the C and O of THF and the O of H_2_O were set as the works of Wu et al. [22,23,24] Interaction parameters between other mixed atoms were generated by standard Lorentz-Berthelot combining rules. The SETTLE algorithm was used to treat water as rigid molecules [49]. The cutoff length of short-range interactions was set to 1.2 nm, and long-range Coulomb interactions were determined using the Fast Smooth Particle Mesh Ewald method (PME) method [50]. Periodic boundary conditions were applied in all three directions. The steepest descent method was used in the energy minimization. A V-rescaling thermostat [51] and Berendsen barostat [52] were used to control the temperature and pressure during simulations, with coupling time constants of 0.2 and 2.0 ps, respectively. Semi-pressure control was used in this work so that the x/y direction and the z direction were scaled independently. The leapfrog algorithm was used in the motion equations with a time step of 2 fs. All MD simulations were performed using GROMACS 2019.3 [53].

As the lifetime of the amorphous cage formed is very short in the initial stage [54,55], a homemade algorithm was used to identify the cage structure during simulations. The structure of the water molecules was identified by those oxygen atoms within 0.62 nm around the oxygen and carbon atoms in THF molecules, and within 0.62 nm around the center of nitrogen molecules. Two oxygen atoms were regarded as being connected with a distance less than 0.35 nm. Then, all the pentagonal and hexagonal rings were identified via connected oxygen atoms. Therefore, the 5^12^, 6^2^5^12^, 6^3^5^12^, and 6^4^5^12^ cages containing THF or N_2_ molecules can be recognized. However, the five-water rings were identified by searching all the water molecules in the whole system. Two water molecules are regarded as being connected by a hydrogen bond, when the r_oo_ (the distance between oxygen atoms) is less than 0.35 nm and ∠OOH (the angle between the O–O vectors and O–H bond) is less than 30°. 

## 3. Results and Discussions

### 3.1. Formation Processes of Hydrates with or without of a Nitrogen Bubble

**Figure 2:**
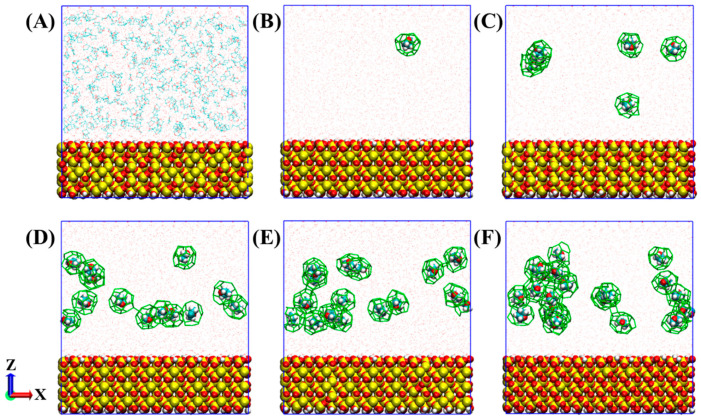
Formation process of hydrates in the system without nitrogen bubbles: (**A**) 0 ns, (**B**) 100 ns, (**C**) 200 ns, (**D**) 400 ns, (**E**) 800 ns, and (**F**) 2000 ns. THFs not in hydrate cages are not represented in (**B**–**F**) for simplicity. Atoms are shown as Figure 1, and THF-filled cages are represented as green stick lines.

As displayed in Figure 2, in systems without a nitrogen bubble, THF-filled cages can be generated randomly in the solution, away from the surfaces of the quartz substrate. With the simulation, the number of THF-filled cages also increases. Besides, some THF-filled cages were found to be connected with each other by sharing faces or forming hydrogen bonds. However, it was found that the THF-filled cages are not stable, and many formed ones were decomposed according to the snapshots. The short lifetime of THF-filled cages may be due to the hydrogen bonding interactions between water and tetrahydrofuran, which is not conducive to the formation of regular cages [24].

**Figure 3:**
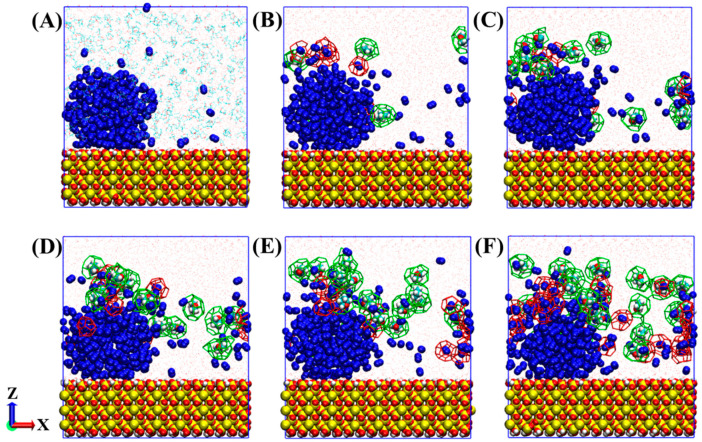
Formation process of hydrates in a system with a nitrogen bubble: (**A**) 0 ns, (**B**) 100 ns, (**C**) 200 ns, (**D**) 400 ns, (**E**) 800 ns, and (**F**) 2000 ns. Isolated THF molecules are not represented in (**B**–**F**) for a clearer display. The atomic representation is the same as in Figure 1. THF and N_2_ cages are represented as green and red stick lines, respectively.

For comparison, Figure 3 displays the formation process of hydrates under the influence of a nitrogen bubble. Similarly, the number of hydrate cages increases with the simulation. The difference is that in the first 800 ns, most hydrate cages formed around the nitrogen bubble and occupy N_2_ molecules. Subsequently, THF-filled cages can be formed either around the nitrogen bubble or in the solution, and it is obvious that the number of THF-filled cages around the nitrogen bubble is much more than that in the solution. In addition, the connectivity of the hydrate cages around bubbles is much better, unlike hydrate cages in solution, which are mostly isolated.

**Figure 4:**
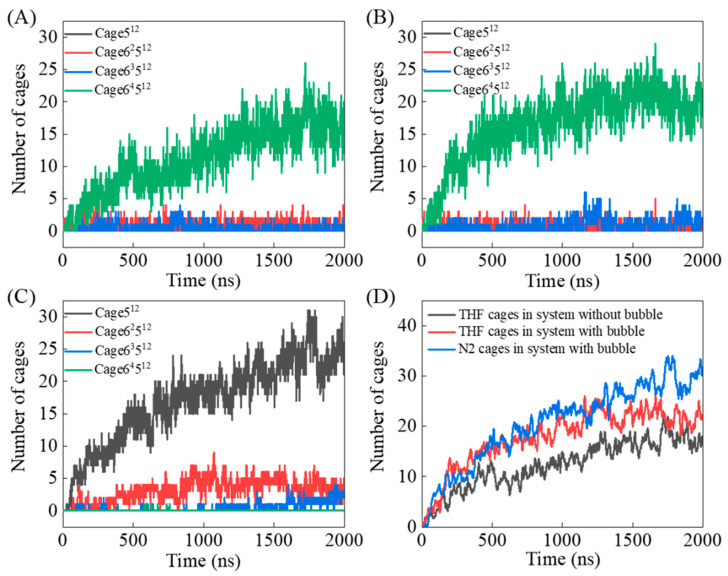
Time evolution of the number of (**A**) THF-filled cages in system without a nitrogen bubble, (**B**) THF-filled cages and (**C**) nitrogen cages in system with a nitrogen bubble, and the total THF-filled cages and nitrogen cages in each system. (**D**) Comparison of cage numbers for systems with or without a nitrogen bubble.

To clarify the mechanism, the evolution trends of the number of 5^12^, 6^2^5^12^, 6^3^5^12^, and 6^4^5^12^ cages containing THF or N_2_ molecules were monitored, as shown in Figure 4. In systems without a nitrogen bubble, THF hydrates can nucleate within several nanoseconds, and the number of THF-filled cages gradually increased (Figure 4A). In systems with the nitrogen bubble, hydrates can also nucleate in a short time, with both THF-filled cages and N_2_-filled cages being formed (Figure 4B,C). During the whole simulation process, the number of THF-filled cages increased rapidly in the first 500 ns, and the growth rate slowed down after 500 ns. As THF molecules are large, they are mainly wrapped in 6^4^5^12^ large cages. Although other irregular THF-filled cages are also formed during the simulation, the numbers of them are small and remained below five (Figure 4A,B). By comparison, it is obvious that the system with bubbles generates more THF-filled cages than the system without bubbles (Figure 4D). In the system with a nitrogen bubble, due to the small size of N_2_ molecules, it can be occupied in the small 5^12^ cage. The filling of nitrogen molecules will reduce the binding energy of the hydrate system, which in turn will increase the stability of the hydrate structure.

**Figure 5:**
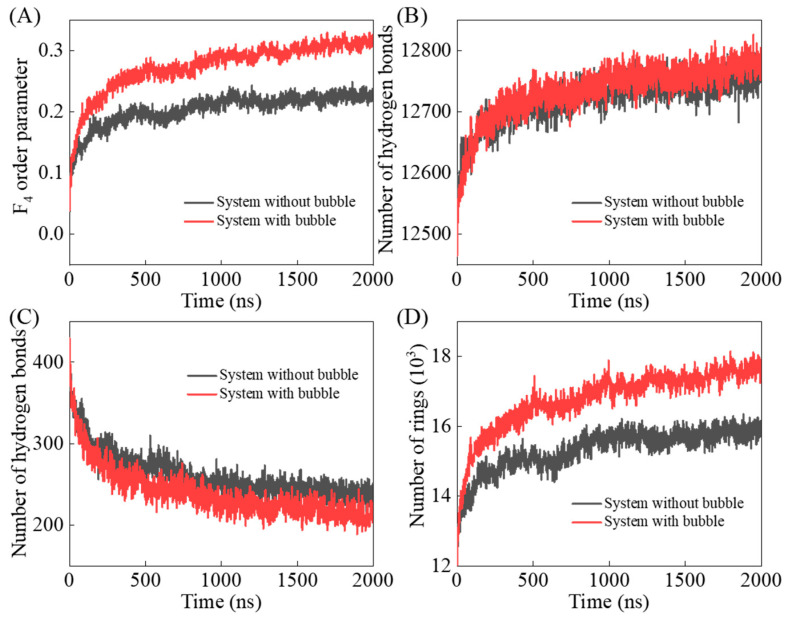
Time evolution of (**A**) F_4_ order parameter, the number of hydrogen bonds between water molecules (**B**) and water and THF molecules (**C**), (**D**) the number of five-water rings.

To better characterize the evolution of hydrate crystal structure, the F_4_ (Four-body) order parameters, the number of hydrogen bonds between water molecules and between water and THF molecules, and the number of five-water rings are calculated (Figure 5). F_4_ order parameters are calculated by analyzing the directivity and saturation of hydrogen bonds, and it should be noted that the F_4_ values for liquid water, ice, and hydrates are −0.04, −0.4, and 0.7, respectively [56]. After performing the relaxation simulation of the two systems with or without a nitrogen bubble, the F_4_ value of the initial configuration was about 0.04. With the simulation, the increase of F_4_ values indicates that the molecular structures of water become more ordered. However, for systems without and with a nitrogen bubble, F_4_ values reached about 0.23 and 0.31 at the end of the simulation, respectively, which means that the existence of the nitrogen bubble promotes the formation of hydrate. However, even at the end of the simulation, the F_4_ value of both systems did not reach 0.7, which is caused by the fact that the hydrate structure do not fill the whole THF solution.

THF molecules have the ability to form hydrogen bonds with water molecules, but the hydrogen bonds between water and THF molecules in hydrates are weak, and only less than 5% of the THF molecules in hydrates are able to form hydrogen bonds with water molecules [57]. It was found that with the formation of THF hydrate, the number of hydrogen bonds between water molecules increased, while the number of hydrogen bonds between water and THF molecules decreased, as shown in Figure 5B,C. In addition, more water–water hydrogen bonds and less water–THF hydrogen bonds were found in the system with a nitrogen bubble, indicating that the THF-filled cages in this system have better stability.

Five-water rings are the basic components of hydrate cages, which is one of the key issues to describe the changes of molecular structure during hydrate formation and to evaluate the influence of nitrogen bubbles. As shown in Figure 5D, a certain number of rings already exist in the initial configuration of the two systems, and there is no significant difference between the number of rings. With the simulation, the number of five-water rings in both systems increased, and there are more water rings in the system with a nitrogen bubble. At the end of simulation, about 17,700 and 15,800 five-water rings existed in the systems with and without the nitrogen bubble, respectively. The simulation shows that nitrogen molecules can be dissolved from nitrogen bubble into the THF solution, and these dissolved nitrogen molecules are the key to induce the formation of more five-water rings.

### 3.2. Effects of the Nitrogen Bubble on Hydrate Formation

**Figure 6:**
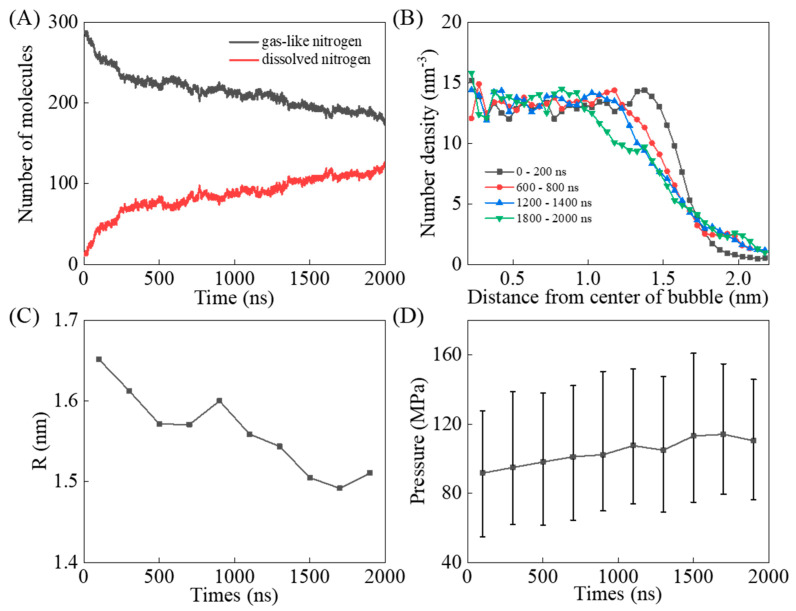
Time evolution of (**A**) nitrogen molecules in different states, (**B**) distribution of N_2_ along the distance from center of bubble, (**C**) average radius of nitrogen bubble in each 200 ns, and (**D**) average inner pressure of the nitrogen bubble in each 200 ns.

Firstly, the evolution of the nitrogen bubble during hydrate crystallization was analyzed. Dissolved nitrogen molecules are defined as those surrounded by more than 16 water molecules within 0.55 nm of the center of the nitrogen molecule. For simplicity, nitrogen molecules occupied in hydrate cages are also regarded as dissolved molecules [58]. As shown in Figure 6A, nitrogen molecules can be dissolved into THF solution from the nitrogen bubble with the simulation, resulting in a decrease in the number of gas-like nitrogen molecules. The nitrogen bubble was simplified into a spherical shape to analyze the distribution of nitrogen molecular number density at different simulation stages, as shown in Figure 6B. The radius of the bubble describes the distance from the bubble center to the point where the gas molecular density reaches half the value in the bulk of the bubble [59,60]. According to this calculation method, the radius of the nitrogen bubble is distributed between 1.5 and 1.7 nm, and shows a downward trend with the continuous dissolution of gas-like nitrogen molecules, as shown in Figure 6C. It was found that the inner pressure of the nitrogen bubble increased with the crystal growth of hydrates, which was also verified by independent simulations of nitrogen molecules with the same number density at the same temperature, as shown in Figure 6D. The increase of the inner pressure of the nitrogen bubble can promote the dissolution of nitrogen molecules into THF solution.

**Figure 7:**
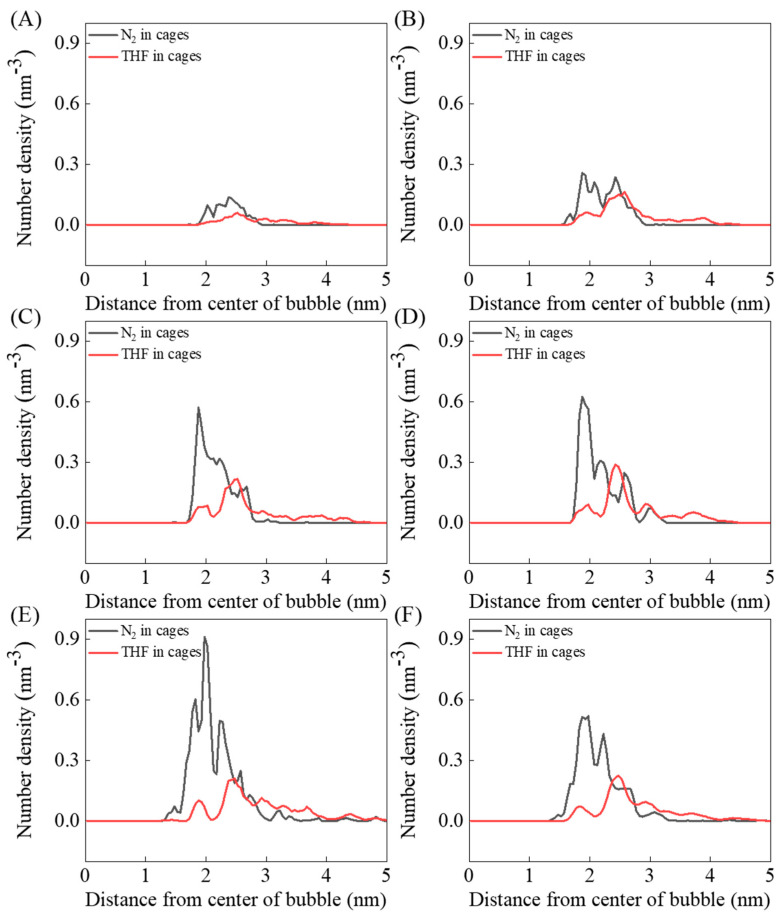
Distribution of caged N_2_ and THF along the distance from center of bubble during (**A**) 0–200 ns, (**B**) 200–400 ns, (**C**) 400–600 ns, (**D**) 600–800 ns, (**E**) 1800–2000 ns, and (**F**) 0–2000 ns.

Many dissolved nitrogen molecules are occupied in hydrate cages, and the formed N_2_-filled cages actually affect the formation and distribution of THF-filled cages, as illustrated in Figure 3. Figure 7 shows the evolution distribution of caged N_2_ and THF molecules with simulation time. In the first 200 ns, most of the caged N_2_ and THF molecules were distributed at about 2.5 nm away from the center of the bubble (1 nm away from the surface of the bubble), and caged N_2_ molecules were closer to the bubble center than caged THF molecules. As mentioned above, the radius of nitrogen bubbles decreased with the simulation, and the subsequent caged N_2_ molecules were mainly distributed at 2 nm from the bubble center, which became closer to the bubble center with the extension of simulation time, as shown in Figure 7B–E. The distribution of THF-filled cages gradually presents three aggregation peaks. The first batch of THF molecules are occupied in hydrate cages about 2 nm away from the bubble center, corresponding to the bubble surface, and some tetrahydrofuran molecules are enclosed in a hydrate cage about 2.5 nm away from the center of the bubble. The above two batches of THF-filled cages are mainly connected with N_2_-filled cages near the bubble surface. The rest are isolated THF-filled cages distributed in the solution, away from the nitrogen bubble.

**Figure 8:**
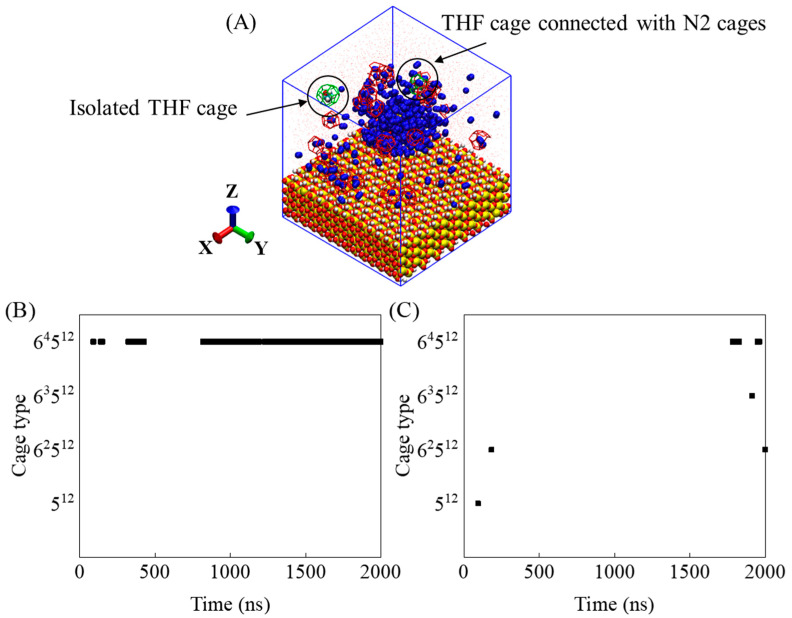
(**A**) Typical THF-filled cage connected with N_2_-filled cages and isolated THF-filled cage, and the evolution of the cage types of (**B**) THF-filled cage connected with N_2_-filled cages and (**C**) isolated THF-filled cage.

Figure 8 shows the results of analysis to further study the effect of nitrogen cages on hydrate stability. Two typical THF-filled cages are shown in the snapshot in Figure 8A. One is a THF-filled cage connected with N_2_-filled cages on the bubble surface, and the other is an isolated THF-filled cage in solution. The evolutions of the above two cages were calculated and shown in Figure 8B,C. It can be seen that when the THF-filled cage is connected with N_2_-filled cages, the THF molecule can be stably enclosed in 6^4^5^12^ cages for a long time. In contrast, the structure of the isolated THF-filled cage is changeable and difficult to stabilize. The short lifetime of isolated THF-filled cages may be because of the hydrogen bond interaction between THF and water molecules [24]. However, the N_2_-filled cages pre-formed in the system can serve as stable sites for hydrate crystal growth, prolonging the lifetime of THF-filled cages in solution.

**Figure 9:**
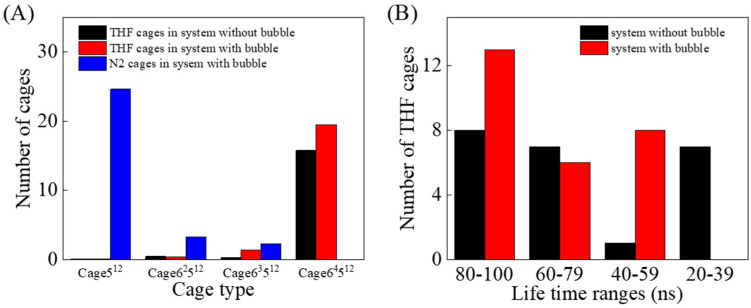
(**A**) The number of THF-filled cages and N_2_ cages, and (**B**) lifetime distribution of THF-filled cages in the last 100 ns.

The number of THF-filled cages and N_2_-filled cages, and the lifetime distribution of THF-filled cages in the last 100 ns of the two simulations were calculated, as displayed in Figure 9. N_2_ molecules are mainly occupied in small 5^12^ cage, while THF molecules are more likely to be enclosed in large 6^4^5^12^ cage, as presented in Figure 4A-C. In the system with a nitrogen bubble, the generation of THF-filled cages was positively correlated with the number of N_2_-filled cages. In addition, in the last 100 ns simulation, the average lifetime of the THF-filled cages in the system with a nitrogen bubble was much longer, which is affected by the promotion of the formation of N_2_-filled bubbles.

## 4. Conclusions

Based on the classical MD simulations of the crystallization of THF hydrates under the effect of nitrogen nanobubble, the bubble can have a promoting effect on hydrate nucleation. Through comparison, it is found that in the system with a nitrogen bubble, more THF-filled cages were formed, the crystal structure was more orderly, more water–water hydrogen bonds and less water–THF hydrogen bonds were formed, and more five-water rings were generated.

The promotion of the nitrogen bubble on hydrate crystallization comes from the dissolution of nitrogen molecules. With the growth of hydrate crystals in the system with a bubble, the inner pressure of the nitrogen bubble increases, resulting in the dissolution of some nitrogen molecules into THF solution and the reduction of bubble radius. Some of dissolved nitrogen molecules can be enclosed in small hydrate cages near the nitrogen bubble. Most of the THF-filled cages formed subsequently are connected to N_2_-filled cages near the bubble surface, and only a few cages are isolated in the THF solution. Moreover, the N_2_-filled cages pre-formed in the system can serve as stable sites for hydrate crystal growth, resulting in the fact that THF-filled cages connected with N_2_ cages are much more stable and with a longer lifetime than isolated cages in solution.

## Figures and Tables

**Table 1:** Interaction Lennard-Jones Parameters between THF and Water [22,23,24].

C_THF_-O_H2O_		O_THF_-O_H2O_	
ɛ (kcal·mol^−1^)	σ (nm)	ɛ (kcal·mol^−1^)	σ (nm)
0.2547	0.3430	0.1509	0.3163

## Data Availability

The data used to support the findings of this study are available from the corresponding author upon request.

## Supplementary Materials

The following supporting information can be downloaded at: https://www.mdpi.com/article/10.3390/molecules27154945/s1, Figure S1. Time evolution of F4 order parameter of three repeated simulations for the systems with bubbles and the simulation for the system without bubbles. Figure S2. A comparative simulation involving only 100 nitrogen molecules. Figure S3. Effects of pressure and temperature on the time evolution of the number of nitrogen molecules in gas phase and F_4_ order parameter in the systems with nitrogen bubbles.

## Sample Availability

Samples of the compounds are not available from the authors.
