# Supplementary material for: Molecular Insights into the Effect of Nitrogen Bubbles on the Formation of Tetrahydrofuran Hydrates
Source: Molecules. 2022 Aug 3;27(15):4945. doi: 10.3390/molecules27154945 (PMC9370114; doi:10.3390/molecules27154945)
Supplement: Supplementary file 1 [file molecules-27-04945-s001.zip › molecules-1809052-supplementary.pdf]

Supporting Information

Molecular Insights into the Effect of Nitrogen Bubbles on the Formation of Tetrahydrofuran Hydrates

Xin Huang [1,3,#], Zhenchao Li [2,#], Le Zhang [1,3], Jiayuan He [1,3] and Hailong Lu [2,*]

[1] SINOPEC Petroleum Exploration and Production Research Institute, Beijing 102206, China;
[2] Beijing International Center for Gas Hydrate, School of Earth and Space Sciences, Peking University, Beijing 100871, China
[3] Southern Marine Science and Engineering Guangdong Laboratory (Guangzhou), Guangzhou 511458, China
[#] These authors contributed equally to this paper
[*] Correspondence: hlu@pku.edu.cn; Tel.: +8618611029001

1. The validation of the simulation

Two repeated simulations were carried out for the system with bubbles, recorded as run2 and run3. The F4 value of each simulation is shown in Figure 1. The time evolutions of F4 order parameter in the two repeated simulations (run2 and run 3) represent similar trend as that in the simulation in original manuscript (run1). Therefore, the results obtained are reliable.

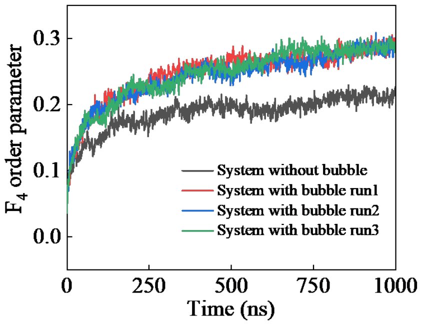

Figure S1. Time evolution of F4 order parameter of three repeated simulations for the systems with bubbles and the simulation for the system without bubbles.

2. The sensitivity analysis of parameters

According to our analysis, the formation of hydrate is mainly affected by the number of nitrogen molecules in the bubble, temperature and pressure. The influence of nitrogen bubbles is the focus of our research. If the nitrogen molecular number of the system is set to be small, the bubbles will dissolve in a short time, which cannot provide enough data for analysis. For your reference, an example of 100 nitrogen molecules simulation is made, as shown in Figure S2. All 100 nitrogen molecules are dissolved into THF solution in 500 ns.

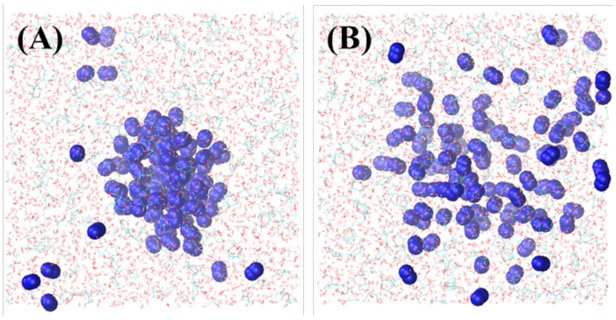

Figure S2: A comparative simulation involving only 100 nitrogen molecules.

In addition, the sensitivity of temperature and pressure is also analyzed, as shown in Figure S3. Comparatively speaking, these two parameters are not sensitive to the influence of hydrate formation rate, but will have a significant influence on the dissolution rate of nitrogen molecules. The sensitivity of temperature and pressure is not the focus of the article, and the related work is not shown in this manuscript.

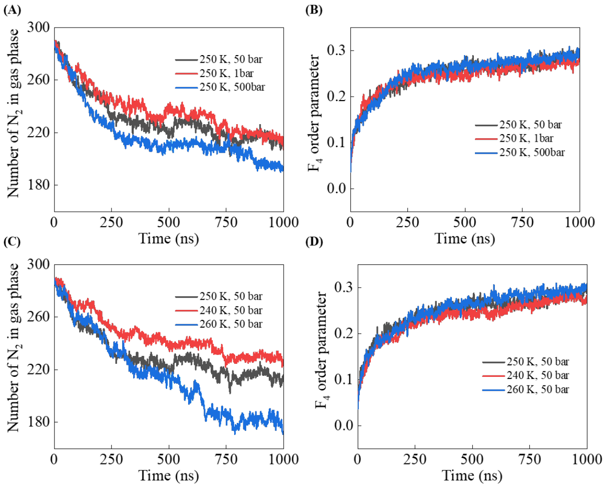

Figure S3. Effects of (A, B) pressure and (C, D) temperature on the time evolution of (A, C) the number of nitrogen molecules in gas phase and (B, D) F4 order parameter in the systems with nitrogen bubbles.

In order to study the effect of nitrogen bubble on THF hydrate formation, a relatively stable bubble needs to be placed in the system. It is difficult to crystallize THF hydrate in molecular simulation, and the system is not stable after 2000ns simulation. After several comparisons, the current simulation conditions are selected. On the one hand, ensure the stability of nitrogen bubbles, on the other hand, provide a greater driving force for promoting the formation of THF hydrate.
